# The genetics of non-syndromic dentinogenesis imperfecta: a systematic review

**DOI:** 10.1007/s40368-024-00992-6

**Published:** 2025-01-13

**Authors:** M. Gilani, A. Saikia, R. Anthonappa

**Affiliations:** Dental School, The University of Western Australia, 17 Monash Avenue, Nedlands, WA 6009 Australia

**Keywords:** Dentinogenesis imperfecta, Hereditary dentine defects, Dentine sialophosphoprotein (DSPP), Collagen type I alpha 1 chain (COL1A1), Collagen type I alpha 2 chain (COL1A2)

## Abstract

**Purpose:**

This systematic review aims to consolidate existing genetic and clinical data on non-syndromic dentinogenesis imperfecta (DI) to enhance understanding of its etiology.

**Methods:**

Electronic databases were searched for genetic familial linkage studies published in English without time restrictions. Genetic familial linkage studies that reported cases of Shield’s classifications: DI-II, DI-III or DD-II were included. After removing duplicates and excluding non-eligible articles, two reviewers screened relevant articles independently, followed by data extraction.

**Results:**

The systematic search identified 3475 articles, with 135 suitable for full-text review and a final 41 that met inclusion criteria. Within this set of studies, 10 conducted a histopathologic examination of teeth from affected participants. *DSPP* mutations were the most frequently reported, with 59 documented mutations. Four studies identified mutations in *COL1A1* and *COL1A2*, revealing non-syndromic DI cases, predominantly in individuals of Asian descent. Histopathological analysis of affected teeth showed variations in pulp chamber size, dentinal tubule irregularities, enamel malformations, and mineral density reductions, depending on DI phenotype.

**Conclusions:**

This review consolidates genetic and clinical data to advance the understanding of non-syndromic DI. It highlights the role of *DSPP*, *COL1A1* and *COL1A2* and the potential involvement of other genes, emphasizing the effectiveness of whole-exome sequencing in identifying causative mutations.

## Introduction

Dentinogenesis imperfecta (DI) is an inherited dental disorder characterized by a profound deficiency in dentine mineralization and structural abnormalities (Barron et al. 2008). The prevalence of DI varies depending on the population studied, ranging from 1 in 6000–8000 births, as reported by Witkop in 1957 (Witkop CJ. 1957). Patients with DI typically exhibit a spectrum of phenotypes, which includes discoloration of teeth ranging from blue to brown, often accompanied by a distinctive translucency (Shields et al. 1973). The primary teeth are typically more severely affected as highlighted by Malmgren et al. in 2004 (Malmgren et al. 2004). Additionally, individuals with DI frequently experience accelerated tooth wear due to enamel detachment from the underlying defective dentine (Barron et al. 2008).

The most widely accepted classification system for hereditary dentine disorders, introduced by Shields et al., broadly categorized into two primary groups based on their clinical manifestations, namely DI and Dentine Dysplasia (DD). DI has three recognized types, labeled as DI type I, II, and III. DD is further divided into two subtypes, DD type I and II, (Shields et al. 1973). The evolution of genetics underlying DI has led to a revised classification system proposed by de La Dure-Molla in 2015. This updated framework differentiates DI into categories based on severity—mild, moderate, and severe and introduces a distinct category for radicular Dentine Dysplasia (radicular DD) (de La Dure-Molla et. al. 2015).

Most DI cases reported in the literature are caused by mutations affecting the dentine sialophosphoprotein (*DSPP)* gene. The *DSPP* gene is responsible for encoding a member of the small integrin-binding ligand N-linked glycoprotein (SIBLING) family of proteins. This gene yields a single mRNA that generates three distinct proteins upon cleavage at specific sites: dentine sialoprotein, dentine glycoprotein, and dentine phosphoprotein, as elucidated by Yamakoshi et al. in 2008 (Yamakoshi et al. 2008). These proteins play crucial roles in dentine mineralization and are instrumental in the transformation of pre-dentine into fully mineralized dentine, a process well-documented by Suzuki in 2009 (Suzuki et. al. 2009). Recent findings have linked mutations in *COL1A1* and *COL1A2* to non-syndromic DI. This suggests that, in future investigations, these gene mutations should be considered as potential contributors to isolated dentine abnormalities, alongside *DSPP *(Lee et al. 2021, Udomchaiprasertkul et al. 2020, and Zeng et al. 2021).

Given the increased focus on genetic and molecular analyses in recent years concerning patients with DI, the specific objectives of this systematic review were to (1) conduct a comprehensive review of all genetic familial linkage studies related to DI in English, focusing on analyzing the association between genetic mutations and DI, and (2) synthesize and integrate clinical and molecular data from published DI literature.

## Materials and methods

This study adhered to the Preferred Reporting Items for Systematic Reviews and Meta-analyses (PRISMA) Statement guidelines, as outlined by Page et al. in 2021 (Page et al. 2021).

To formulate the research questions for this study, the PECOS acronym was employed:

- P (Participants): Patients diagnosed with DI.
- E (Exposition): The examination of mutational analysis and the clinical, radiographic, or histopathologic dental characterization of DI.
- C (Comparison or Control): Cases involving the absence of DI for comparison.
- (Outcome Measures): The investigation of the prevalence of genetic variants in patients with *DSPP* mutations associated with DI, the prevalence of genetic variants in patients with *COL1A2* mutations related to DI, the prevalence of genetic variants in patients with *COL1A1* mutations linked to DI, and the identification of any genetic hotspots that can predict the occurrence of DI.
- S (Types of Included Studies): This study encompassed genetic linkage analysis studies, observational studies, case reports, and case series.

### Search strategy and selection criteria

The systematic review was registered on the PROSPERO database (CRD42023470897). In April 2023, an electronic search was conducted across several databases, namely PubMed/MEDLINE, Web of Science, ScienceDirect, and Scopus, without any time restrictions. The search strategies employed the terms 'dentinogenesis imperfecta' OR 'dentine/dentin dysplasia.' A gray literature search using Google Scholar was also carried out with the same search terms. A manual search was conducted in all relevant dental and genetic journals. To identify potential additional studies, the reference lists of the identified studies and pertinent reviews on the subject were also examined. The survey encompassed articles published on or before April 2023.

To ensure the elimination of duplicate references, EndNote® X7 reference manager software was utilized. Subsequently, the de-duplicated dataset was managed and processed using Rayyan^®^ (Ouzzani et al. 2016).

### Inclusion and exclusion criteria

Criteria for article inclusion were:

- For this review, studies that reported cases of Shield’s classifications: DI-II, DI-III or DD-II were included. This choice ensures that the research encompasses a broad spectrum of DI manifestations, allowing for a comprehensive understanding of the genetic and phenotypic variations.
- Investigations presenting comprehensive elucidation of variations within the *DSPP, COL1A1,* or *COL1A2* genes.
- Reports with comprehensive clinical, radiographic, or histopathological assessments of DI.

Criteria for the exclusion of articles were:

- Studies without molecular analyses and reporting cases of DI-I.
- Investigations with ambiguous or deficient DI diagnoses and a paucity of substantial clinical, radiographic, or histopathological details.
- Instances involving patients concurrently affected by systemic conditions, such as osteogenesis imperfecta (OI).
- Research studies addressing dental abnormalities unrelated to DI.
- Experimental studies conducted either in vitro or in vivo.
- Publications not documented in the English language.

### Study selection

Titles and abstracts of all reports identified reports from the electronic searches underwent independent review by two authors, MG and RA. The full-text articles were obtained in cases where the studies seemed to align with the inclusion criteria or if the title and abstract lacked sufficient data for a definitive decision. Any disparities in judgment were deliberated upon until a consensus was achieved.

### Data extraction

Two authors, MG and RA, gathered pertinent information from each selected article. A third author (AS) checked the information. Any disagreements were discussed until a consensus was reached among the three authors (MG, RA, AS). The extracted data encompassed details, such as study design, geographical location of the study, ethnicity of the patients, categorization of the condition based on the clinical grading system proposed by Shields, the sequencing methodology employed, and the identification and characterization of mutations in the DSPP gene or other genes potentially associated with the pathogenesis of DI.

## Results

### Literature search

**Fig. 1:**
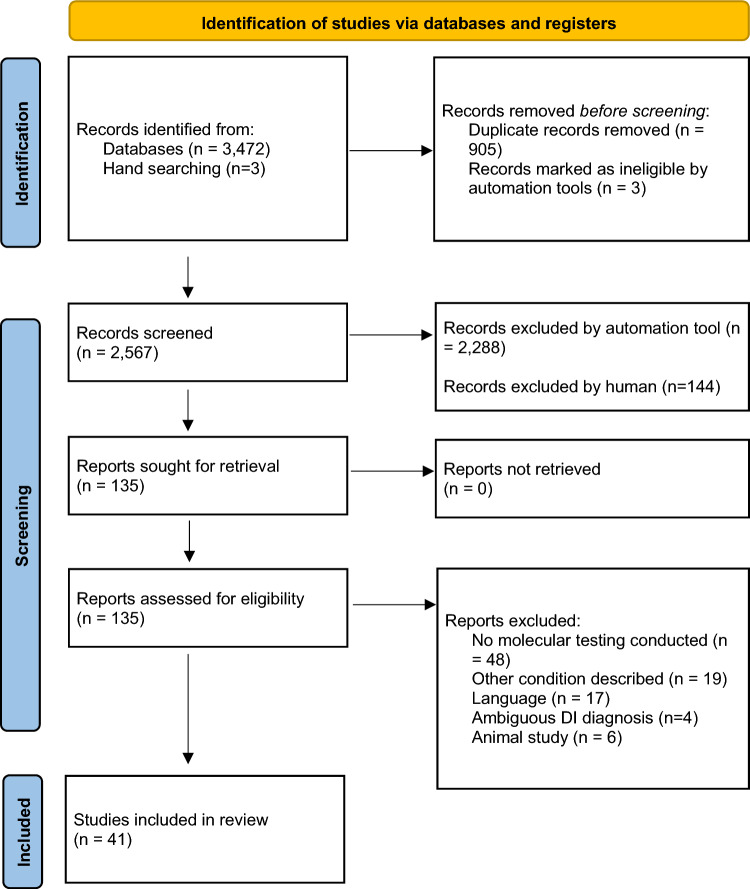
Process of data collection in accordance with the PRISMA statement for systematic reviews

The study selection process is outlined in Fig. 1. Initially, 3475 articles were identified through a combination of database and manual searches. After identifying and removing duplicate citations and ineligible studies, 135 articles were assessed for inclusion based on their titles and abstracts. Further analysis of the full-text articles resulted in 94 articles being further excluded. Ultimately, 41 publications met the inclusion criteria. Within this set of 41 family linkage analysis studies, ten studies conducted a histopathologic examination of teeth from affected participants.

### Description of the studies and analyses

Table 1 provides a comprehensive breakdown of *DSPP COL1A1, COL1A2* gene mutations found in the literature, along with the corresponding case counts, ethnicity and the clinical classification of the disease.

**Table 1:** Summary of reported mutations within *DSPP, COL1A1, COL1A2* gene causing inherited dentine defects (Dentinogenesis Imperfecta)

Location	Total number of reported mutations	Total number of affected individuals	Reported mutation category	Ethnicity	cDNA mutation	Protein change	Number of affected individuals reported *(n)*	Age range	Phenotype	References
Reported mutations within DSPP gene causing DI										
Exon 2	5	47	Missense (*n* = 31)	American (US) (*n* = 8) Asian (Thailand, China, Korea, Taipei, Taiwan, Turkey) (*n* = 27) European (Sweden, Copenhagen, UK) (*n* = 10)	c.16 T > G	p.Y6D	7	Unclear	DD-II	Rajpar et al. 2002
					c.44C > T	p.A15V	8	1.5–48 yrs	DI-II	Malmgren et al. 2004
					c.49C > A	p.P17T	5	20–72 yrs	DI-II	Xiao et al. 2001
					c.49C > T	p.P17S	9	Unclear Unclear 1.5–11.5 yrs	DI-II	Zhang et al. 2007, McKnight et al. 2008a, Taleb et al. 2018
					c.50C > T	p.P17L	18	Unclear 2.8–29.7 yrs 7–38 yrs 3–38 yrs	DI-II	Li et al. 2012, Lee et al. 2013, Porntaveetus et al. 2019, Simmer et al. 2022
Intron 2	7	56	Splice site (*n* = 56)	American (USA) (*n* = 1) Asian (Korea, Taiwan, Taipei, China) (*n* = 31) European (Copenhagen, Turkey) (*n* = 58)	c.52-3C > G	p.V18_Q45del	6	22 mo–4 yrs	DI-II	Kim et al. 2004
					c.52-3C > A		20	Unclear	DI-II	Holappa et al. 2006
					c.52-6 T > G	p.V18_Q45del	1	20–72 yrs	DD-II	Lee et al. 2008
					c.52-25del23bp		24	1–65 yrs	DI-II	Wang et al. 2009
					c.52-2A > G	p.V18_Q45del	2	unclear 1.5–11.5 yrs	DI-III (*n* = 1) DI-II (*n* = 1)	Li et al. 2017, Taleb et al. 2018
					c.52-2Adel	p.V18_Gln45del	1	4–18 yrs	DI-II	Kim et al. 2022
					c.52 − 1G > A		2	3–38 yrs	DI-III	Simmer et al. 2022
Exon 3	7	55	Missense (*n* = 39) Nonsense (*n* = 18)	American (USA) (*n* = 1) Asian(China, Japan, Korea) (*n* = 57) European (Copenhagen, Turkey) (*n* = 5)	c.52G > T	p.V18_Q45del or p.V18F	13	20–72 yrs 22 mo–4 yrs 1–82 yrs Unclear Unclear	DI-II (*n* = 11) DI-III (*n* = 2)	Xiao et al. 2001, Kim et al. 2005, Song et al. 2006, Holappa et al. 2006, Li et al. 2017
					c.133C > T	p.Q45X	18	Unclear 1–82 yrs	DI-II	Zhang et al. 2001, Song et al. 2006
					c.53 T > A	p.V18D	18	2.5–4.5 yrs 4–35 yrs	DI-II/III (*n* = 15) DI-II (*n* = 3)	Lee et al. 2009, Kida et al. 2009, Lee et al. 2011a
					c.53 T > G	p. Val18Gly	2	7–40 yrs	DI-II	Du et al. 2021
					c.135G > T	p.Gln45His	1	1.5–11.5 yrs	DI-II	Taleb et al. 2018
					c.135G > A		1	4–18 yrs	DD-II	Kim et al. 2022
					c.53 T > C	p.Val18Ala	2	3–38 yrs	DI-III	Simmer et al. 2022
Intron 3	5	62	Splice site (*n* = 62)	American (USA) (*n* = 3) Asian (China, Korea, Taiwan, Taipei) (*n* = 57) European (Turkey) (*n* = 2)	c.135 + 1G > A	p.V18_Q45del	19	20–72 yrs	DI-II	Xiao et al. 2001
					c.135 + 1G > T	p.V18_Q45del	1	Unclear	DI-II	McKnight et al. 2008a
					c.135 + 1G > C		1	4–18 yrs	DI-III	Kim et al. 2022
					c.135 + 2 T > C		16	Unclear	DI-II	Zhang et al. 2011
					c.135 + 3A > G		25	5–32 yrs 3–38 yrs	DI-II (*n* = 22) DI-III (*n* = 3)	Bai et al. 2010, Simmer et al. 2022
Exon 4	1	9	Missense (*n* = 9)	European (Sweden, Finland) (*n* = 9)	c.202A > T	p.R68W	9	1.5–48 yrs	DI-II	Malmgren et al. 2004, Holappa et al. 2006
Exon 5	34	80	Frameshift (*n* = 38) Insertion/Deletion (*n* = 10)	American (USA) (*n* = 23) African (Madagascar, Africa)(*n* = 9) Asian (China, Thailand, Korea) (*n* = 27) European(Turkey) (*n* = 22)	c.1870_1873delTCAG	p.S624TfsX687	1	Unclear	DD-II	McKnight et al. 2008a
					c.1918_1921delTCAG	p.S640TfsX673	6	Unclear 3–40 yrs 3–38 yrs	DD-II	McKnight et al. 2008a, Nieminen et al. 2011, Simmer et al. 2022
					c.2272delA	p.S758AfsX554	1	Unclear	DI-II	McKnight et al. 2008a
					c.2525delG	p.S842TfsX471 p.Ser842Thrfs*472	4	Unclear 3–38 yrs	DI-II	McKnight et al. 2008a, Simmer et al. 2022
					c.3599-3634del36bp c.3715-3716ins18bp	Del1160-1171 Ins1198-1199	10	Unclear	DI-III	Dong et al. 2005
					c. 1686delT	p.D562EfsX752	1	3–40 yrs	DD-II	Nieminen et al. 2011
					c. 1830delC	p.S610RfsX704	1	3–40 yrs	DD-II	Nieminen et al. 2011
					c. 1922-1925delACAG	p.D641AfsX672	1	3–40 yrs	DD-II	Nieminen et al. 2011
					c. 2063delA	p.D688VfsX626	2	3–40 yrs	DD-II	Nieminen et al. 2011
					c. 2349delT	p.S783RfsX531	1	3–40 yrs	DI-II	Nieminen et al. 2011
					c. 2666delG	p.S889TfsX425	1	3–40 yrs	DI-II	Nieminen et al. 2011
					c. 3582-3591delCAGCAGCGAT	p.D1194EfsX117	1	3–40 yrs	DI-II	Nieminen et al. 2011
					c. 3625-3700del76 bp	p.D1209AfsX80	1	3–40 yrs	DI-II	Nieminen et al. 2011
					c.2040delC	p.S680fsX1313	1	Unclear	DD-II	Song et al. 2008
					c.2593delA	p.S865fsX1313	1	Unclear	DI-II	Song et al. 2008
					c.2684delG	p.S895fsX1313	2	Unclear Unclear	DI-II	Song et al. 2008, Li et al. 2017
					c.2688delT	p.S895fsX1313	2	4.5–8.5 yrs	DI-II	Lee et al. 2011b
					c.3135delC	p.S895fsX1313 p.Ser1045Argfs*269	7	Unclear 3–38 yrs	DD-II (*n* = 2) DD-II / DI-II (*n* = 5)	McKnight et al. 2008b, Simmer et al. 2022
					c.3438delC	p.D1146fsX1313	1	Unclear	DI-II	Song et al. 2008
					c.3546_3550del TAGCAinsG	p.D1182fsX1312	1	Unclear	DI-II	Song et al. 2008
					c.3560delG	p.D1182fsX1312	1	4.5–8.5 yrs	DI-II	Lee et al. 2011b
					c.3676delA	p.Ser1226Alafs*88	9	Unclear		Bloch-Zupan et al. 2016
					c.1915_1918delAAGT	p.K639QfsX674 p.Lys639Glnfs*674	4	Unclear 3–38 yrs		Porntaveetus et al. 2018, Simmer et al. 2022
					c.2134delA	p.Ser712Alafs*602	4	Unclear 3–38 yrs	DD-II	Lee et al. 2019, Simmer et al. 2022
					c.3509-3521del13bp	p.D1170AfsX139	1	Unclear	DI-II	Li et al. 2017
					c.1874-1877delACAG	p.D625AfsX687	1	Unclear	DD-II	Li et al. 2017
					c.2833delA		1	22–34 yrs	DI-II	Zhang et al. 2023
					c.2852delG		1	22–34 yrs	DI-II	Zhang et al. 2023
					c.3239delA		1	22–34 yrs	DD-II	Zhang et al. 2023
					c.2035delA	p.S679Vfs*635	1	3–10 yrs	DD-II	Yuan et al. 2023
					c.1871_1874del	p.Ser624fs	2	Unclear	DD-II	Du et al. 2023
					c.3504_3508dup	p.Asp1170Alafs*146	1	5.5–7 yrs		Yang et al. 2016
					c.3461delG	p.(Ser1154MetfsTer160)	6	3–38 yrs	DI-II	Simmer et al. 2022
					c.3700delA		1	3–38 yrs	DD-II/DI-II	Simmer et al. 2022
Reported mutations within COL1A1 gene causing DI										
Exon 22	1	1	Missense	Asian (China)	c.G1463C	p.G488A	1	Unclear	DI-I	Zeng et al. 2021
Reported mutations within COL1A2 gene causing DI										
Exon 19	1	2	NR	Asian (Thailand)	c.1009G > A	p.Gly337Ser	2	17 yrs	DI	Udomchaiprasertkul et al. 2020
Exon 21	1	9	Missense	Asian (Thailand, Korea)	c.1171G > A	p.Gly391Ser	9	22–49 yrs 3–37 yrs	DI-I	Kantaputra et al. 2018, Lee et al. 2021
Exon 48	1	3	NR	Asian (Korea)	c.3233G > A	p.Gly1078Asp	3	3–37 yrs	NR	Lee et al. 2021

Fifty-nine mutations have been reported within the DSPP gene from 37 publications (Table 1). Of these 37 studies, 22% (*n* = 8) used WES to detect *DSPP* mutations, with the earliest report of WES to detect genetic variants in DI from 2016 (Bloch-Zupan et al. 2016). Two hundred and sixty three mutation categories were specified. Of these, 45% (*n* = 118) were splice-site mutations, and 30% (*n* = 79) were missense mutations. Nonsense and insertion/deletion mutations accounted for 7% (*n* = 18) and 4% (*n* = 10) respectively. All frameshift mutations (*n* = 38) were found within exon 5 of the DSPP gene. In 58% of the reported mutations (34/59), *DSPP* mutations were identified in exon 5.

Of the 41 included publications, four studies identified variants in genes outside of *DSPP*, all of which used WES techniques. Fifteen patients from 6 families were identified with mutations in *COL1A1* and *COL1A2,* leading to phenotypes of DI. All reported mutations were identified in those of Asian descent.

Ten studies conducted histopathologic assessments of teeth from individuals affected by the condition (Table 2). Among these studies, 80% (*n* = 8) focused on teeth displaying the DI-II phenotype. Across all studies, irregular dentinal tubules were consistently observed, showcasing variations in their number, shape, and size. Additionally, reductions in microhardness and mineral density were commonly documented.

**Table 2:** Genetic linkage studies reporting histopathologic characteristics and genetic variants associated with dentinogenesis imperfecta

Author, year, country	Teeth (*n*)	Dentition type	Test	Histopathologic Characteristics	Phenotype	Genetic mutation
Malmgren et al. 2004, Sweden	24	Both (Primary, *n* = 16, Permanent, *n* = 8)	Micro-CT	NR	DI-II	Family A: Arg68Trp missense mutation Family B: Ala15Val missense mutation
			Microhardness	NR		
			SEM	NR		
			EDS	NR		
			Histological sections	Severe dysplastic changes		
Song et al. 2006, China	NS	NS	Micro-CT	NR	DI-II	Family 1: nonsense mutation (c.133C-T) Family 2: missense mutation (c.52G-T)
			Microhardness	NR		
			SEM	Irregular, smaller and reduced dentine tubules Lack of scalloping of DEJ		
			EDS	NR		
			Histological sections	NR		
			TEM	Bundles of collagen fibers located around dentinal tubules		
Kida et al. 2009, Japan	NS	Primary	Micro-CT	NR	DI-I	Mutation analysis revealed a mutation (c.53T > A, p.V18D, g.1192 T>A) involving the second nucleotide of the first codon
			Microhardness	NR		
			SEM	NR		
			EDS	NR		
			Histological Sections	No obvious malformation in enamel Reduced, poorly oriented, irregular and widened dentine tubules		
Taleb et al. 2018, Denmark	NS	NS	Micro-CT	NR	DI-II	Family A: missense mutation involving a C-T transition at nucleotide 49 in exon 2 (c.49C >T) Family B: G–T transition at nucleotide 135 in exon 3 (c.135G >T) Family C: A–G transition at c.52-2 in an acceptor splice site of intron 2 (c.52-2 A>G)
			Microhardness	NR		
			SEM	NR		
			EDS	NR		
			Histological Sections	Dysplastic changes (abnormal granular dentine matrix, irregular and reduced dentine tubules, larger size) Lack of scalloping of DEJ		
			Optical Coherence Tomography	Delineated areas of hypomineralization adjacent to DEJ		
Porntaveetus et al. 2018, Thailand	NS	Primary	Micro-CT	Irregular enamel surface Calcified mass in pulp chamber Reduced mineral density in the DGI dentin	DI-I	Mutation analysis revealed a novel heterozygous 4-bp deletion, c.1915_1918delAAGT (p.K639QfsX674)
			Microhardness	NR		
			SEM	Amorphous dentine without dentinal tubules		
			EDS	NR		
			Histological Sections	Scattered, short, irregular and reduced dentinal tubules		
			Cell Proliferation Assay	Cells isolated from dental pulp tissue unable to mineralize		
Porntaveetus et al. 2019, Thailand	NS	NS	Micro-CT	NR	DI-II	Heterozygous pathogenic missense mutation, c.50C > T, p.P17L, in exon 2 of the *DSPP* gene, *DSPP* cells showed deviated mRNA levels of OCN, ALP, and COL1
			Microhardness	NR		
			SEM	Altered cellular morphology and spreading of alveolar bone cells		
			EDS	NR		
			Cell proliferation assay	Colonies formed by DSPP mutant cells slightly lower in number		
Du et al. 2021, China	NS	Primary	Micro-CT	NR	DI-II	Novel heterozygous mutation c.53 T > G (p. Val18Gly) in *DSPP*
			Microhardness	NR		
			SEM	Irregular in size and shape, reduced dentine tubules		
			EDS	Increase in Ca		
			Histological sections	NR		
Zeng et al. 2021, China	NS	NS	Micro-CT	Bulbous shape and color change Obliterated pulp chamber and pulp stones Reduced mineral density	DI-I	A missense mutation (c.1463G > C) in exon 22 of the *COL1A1* gene
			Microhardness	Reduced dentine hardness Reduced elastic modulus		
			SEM	Enlarged, malformed and reduced dentine tubules		
			EDS	Decrease in P No difference in Na, Mg and Ca		
			Histological Sections	NR		
Zhang et al. 2023, China	1	Permanent	Micro-CT	Reduced density of dentine	DI-II	Frameshift mutations (NM_014208.3:c.2833delA, c.2852delG and c.3239delA)
			Microhardness	Reduced microhardness of dentine		
			SEM	Sparse with disordered arrangement Abnormal DEJ		
			EDS	NR		
			Histological sections	NR		
Du 2023, China	4	Primary	Micro-CT	Severe root obliteration, smaller root canals	DD-II	Frameshift deletion mutation c.1871_1874del (p.Ser624fs) in *DSPP*
			Microhardness	Reduced microhardness of dentine		
			SEM	Irregular and reduced dentine tubules		
			EDS	Significant decrease in Mg Significant increase in Na		
			Histological sections	NR		

*DEJ* dentino-enamel junction, *SEM* scanning electron microscopy, *TEM* transmission electron microscopy, *EDS* energy-dispersive spectroscopy, *NR* not reported, *NS* not specified

## Discussion

The objective of this study was to consolidate the existing molecular data related to DI as reported in the literature. Within the scope of this research, we identified and reviewed a total of 322 documented cases of individuals with DI.

Mutations within the *DSPP* gene have a profound impact on the development of both enamel and dentine, as DSPP is expressed by odontoblasts and temporarily in pre-ameloblasts (Bègue-Kirn et al. 1998). The *DSPP* gene is composed of five exons and four introns and encodes a precursor protein that undergoes cleavage, resulting in the production of two mature proteins: DSP and DPP. Exons 1–4 are responsible for encoding DSP, while exon 5 encodes both the carboxy terminus of DSP and DPP. DSP and DPP play vital roles as non-collagenous components within dentine and are crucial for the mineralization process. Research involving mutant mice has demonstrated that DSP primarily regulates the initiation of dentine mineralization, whereas DPP is associated with the maturation phase of dentine mineralization (Suzuki et al. 2009).

Mutations occurring outside the DSPP gene have been documented in a limited number of cases involving non-syndromic DI, specifically in *COL1A1* and *COL1A2*. These genes are responsible for encoding type I collagen, the primary structural component of pre-dentine, comprising approximately 90% of its composition. Beyond its pivotal role in dentine, type I collagen is a fundamental component in various tissues, including bone, tendons, and skin. Consequently, mutations in *COL1A1* and *COL1A2* can result in dentine anomalies seen in syndromes like OI. However, recent research findings have identified mutations in the *COL1A1* and *COL1A2* genes in patients with non-syndromic DI, devoid of any OI-related characteristics. These discoveries indicate that these genes may serve as notable mutational hotspots associated with non-syndromic DI.

In Table 1, the mutations span different exons and introns, leading to a range of protein alterations and disease manifestations. For instance, mutations in Exon 2 include c.16 T > G (p.Y6D) and c.44C > T (p.A15V), with reported cases numbering 7 and 8, respectively, and are associated with developmental disorders (DD-II) and developmental impairments (DI-II). Notably, the c.50C > T mutation (p.P17L) is prevalent with 18 cases and consistently linked to DI-II, as highlighted in several studies including those by Li et al. (2012) and Simmer et al. (2022). Intron 2 mutations, such as c.52-3C > G and c.52-25del23bp, also demonstrate significant variability in phenotypic outcomes. The c.52-2A > G mutation, causing p.V18_Q45del, affects two individuals with DI-III and DI-II, reflecting a diverse range of developmental impairments. Mutations in Exon 3, such as c.133C > T (p.Q45X) and c.53 T > A (p.V18D), are also well-documented with 18 affected individuals respectively and are associated with developmental impairments of both DI-II and DI-III types. Exon 5 mutations, including c.1918_1921delTCAG and c.2525delG, predominantly result in developmental disorders (DD-II and DI-II), with multiple studies documenting these variants in various populations. The data reveal that Exon 2 mutations, primarily missense types, affect individuals across American, Asian, and European populations, with a notable prevalence in Asia. Intron 2 mutations, predominantly splice-site changes, are most common in European populations, with significant representation in Asia as well. Exon 3 mutations, including both missense and nonsense types, are predominantly found in Asian individuals. Intron 3 mutations, all splice-site variants, are heavily concentrated in Asia. Exon 5 mutations, including frameshift and insertion/deletion types, affect a broad range of populations, indicating widespread genetic impact across continents. A single case of a c.G1463C mutation in Exon 22, resulting in a p.G488A protein change, has been reported. This missense mutation was observed in an Asian individual and is associated with a developmental impairment phenotype classified as DI-I. The findings are detailed in the study by Zeng et al. (Zeng et al. 2021) The data reveal several mutations found in Asian populations with specific regional distributions. The c.1009G > A mutation in Exon 19 (p.Gly337Ser), linked to developmental impairment (DI), was observed in 2 cases in Thailand (Udomchaiprasertkul et al. 2020). The c.1171G > A mutation in Exon 21 (p.Gly391Ser), associated with developmental impairment type I (DI-I), was reported in 9 individuals from Thailand and South Korea (Kantaputra et al. 2018; Lee et al. 2021). The c.3233G > A mutation in Exon 48 (p.Gly1078Asp) was noted in 3 cases from South Korea though its specific phenotype is not categorized (Lee et al. 2021). These findings provide a clearer understanding of the mutation distributions and their phenotypic associations in different Asian regions.

Mutations in genes other than *DSPP, COL1A1* and *COL1A2* may cause non-syndromic DI. However, such genetic mutations are yet to be uncovered. In these cases, WES is an ideal genetic testing approach to identify causative mutations in other genes. The relationship between specific genetic mutations and their phenotypic manifestations in DI is complex and crucial for both clinical management and research. The DSPP gene, which encodes the DSPP, plays a central role in dentine formation. Mutations in DSPP lead to various forms of DI, each with distinct clinical and histological features. For instance, in cases of Exon 2, mutation predominantly associated with DI-II, impacted the protein structure, and disrupted dentine matrix formation, leading to softer and more brittle dentine. Clinically, patients with these mutations may experience severe tooth discoloration and fragility, necessitating proactive dental care and intervention. Similarly, Exon 5 mutation leads to frameshift changes, often resulting in severe DI forms. These mutations are linked to significant structural abnormalities in dentine, which are observed as irregular dentinal tubules and compromised mineralization. Clinicians should anticipate these severe manifestations and prepare for more intensive dental management strategies. The identification of non-DSPP mutations in *COL1A1* and *COL1A2* suggests a broader genetic basis for DI beyond DSPP. For example, mutations in *COL1A1* and *COL1A2* can result in dentine anomalies similar to those seen in OI. This finding underscores the importance of considering a broader genetic screening panel when diagnosing DI, particularly in patients with atypical presentations. Non-DSPP DI, namely COL1A1 and COL1A2 mutations, can exhibit a broader range of phenotypic presentations, sometimes overlapping with OI symptoms, but without the full clinical spectrum of OI. Histopathological differences, such as variations in pulp chamber morphology and the extent of pulp obliteration, further distinguish non-DSPP cases from DSPP-related DI.

Microhardness test results indicated that the dentine hardness of DI teeth was lower than that of healthy teeth. This decrease in hardness can be attributed to *DSPP* mutations, which result in the formation of dentine with structural and hardness defects. Scanning electron microscopy results revealed teeth with reduced and irregular tubules. Energy-dispersive X-ray spectroscopy analyses (Du et al. 2021, 2023 and Zeng et al. 2021) showed changes in phosphorus, magnesium, sodium and calcium compared to healthy controls, confirming impaired mineralization of DI dentine. The abnormalities in dentinal structure and elemental content often lead to premature tooth fractures and periapical infections. Abnormal pulp morphology can further complicate dental treatment. Therefore, comprehensive, long-term dental management is of paramount importance for individuals with DI.

The higher portion of Asians could be due to both geographic and sampling tendencies. Geographically, certain DI mutations may be more prevalent in Asian populations, aligning with regional genetic variability. Additionally, enhanced diagnostic capabilities and awareness in Asian countries might lead to more frequent detection and reporting of DI cases. Sampling tendencies could also play a role as majority of the studies included in the review are from Asian regions or if there's a publication bias favoring studies from these areas. To fully understand this discrepancy, it's essential to examine both regional prevalence data and potential biases in study selection and reporting. The histological differences according to genotype in DI highlight how specific genetic mutations impact the dentine structure and formation. DSPP-related DI typically shows more consistent and severe histological abnormalities, while non-DSPP DI (especially with *COL1A1* and *COL1A2* mutations) may present with a broader range of histological variations and additional features related to systemic conditions like OI.

The critical missing element in the current studies is the detailed connection between genotype and phenotype, specifically how mutations in different exons of the DSPP gene manifest both clinically and histologically. While the studies provide valuable information on the types of mutations present in various exons, they often fall short of correlating these genetic variations with specific clinical features and histopathological findings. For instance, a mutation in Exon 2 impacts the dentine’s structure such as tubule morphology or mineral density. However, the translation of these findings to clinical symptoms, such as tooth discoloration or sensitivity, remains unexplained. Without this direct linkage, clinicians lack crucial insights into how these mutations affect disease progression and presentation, impeding the development of targeted diagnostic and therapeutic approaches. Addressing this gap requires more integrated studies that correlate genetic data with detailed clinical and histological outcomes.

Shields’ classification remains a staple in clinical practice due to its established framework, ease of use, and direct applicability. While de La Dure-Molla’s classification provides valuable insights into the genetic aspects of DI, Shields' system offers a more practical approach for everyday clinical decision-making (de La Dure-Molla et. al. 2015, Shields et al. 1973). Among the 41 studies included, 37 followed Shield’s classification, 3 were ambiguous, and 1 utilized the Modified Shield’s classification.

The current study has its limitations. The varying criteria classifying DI across included studies, can impact the comparability of results and the overall synthesis of data. The reliance on studies published in English and the exclusion of experimental studies might limit the diversity of data, potentially skewing results. Furthermore, the inclusion of only well-documented cases with clear DI classification could result in the omission of relevant studies with less definitive diagnoses. The data predominantly focus on studies from specific regions, which may not fully represent global genetic diversity. The concentration of data from Asian populations, for example, might introduce regional biases in understanding mutation prevalence and phenotypic expressions. While the chosen methodology provides a comprehensive overview of DI-related genetic variants, the inherent limitations of classification variability, study biases, and regional focus must be considered. Future research should aim to standardize classification criteria, include diverse populations, and leverage advanced genetic sequencing techniques to enhance the understanding of DI and improve patient management strategies.

Additionally, understanding the genotype–phenotype interactions in DI is critical for advancing clinical practice and improving patient care. By integrating comprehensive genetic testing into clinical practice, utilizing advanced sequencing technologies, and addressing the limitations of current studies, clinicians can enhance diagnosis, prognosis, and management of DI. The accumulated variant data analysis in this systematic review provides a further basis for increasing our comprehension to better predict the occurrence of DI and appropriate patient management. Future research should focus on elucidating the molecular mechanisms of DI, expanding genetic screening, and standardizing diagnostic criteria to improve patient outcomes and guide personalized treatment approaches particularly those related to the expression of *DSPP, COL1A1* and* COL1A2.*

## Conclusion

- Genetic variants within the *DSPP*, *COL1A1* and *COL1A2* genes have been implicated in non-syndromic DI-II, -III and DD-II.
- Most familial genetic linkage studies have conducted single gene analysis, with more recent studies utilizing next-generation sequencing techniques.
- Additional genetic linkage studies are essential in non-syndromic DI-affected families to gain better understanding of its genetic diversity and the potential contribution of other genes.

## References

[CR1] Bai H, Agula H, Wu Q, Zhou W, et al. A novel DSPP mutation causes dentinogenesis imperfecta type II in a large Mongolian family. BMC Med Genet. 2010;11(1):1–6. 10.1186/1471-2350-11-23. 20146806 10.1186/1471-2350-11-23 PMC2829541

[CR2] Barron MJ, McDonnell ST, Mackie I, Dixon MJ. Hereditary dentine disorders: dentinogenesis imperfecta and dentine dysplasia. Orphanet J Rare Dis. 2008;3:1–10. 10.1186/1750-1172-3-31. 19021896 10.1186/1750-1172-3-31 PMC2600777

[CR3] Bègue-Kirn C, Krebsbach PH, Bartlett JD, Butler WT. Dentin sialophosphoprotein, dentin phosphoprotein, enamelysin and ameloblastin: tooth-specific molecules that are distinctively expressed during murine dental differentiation. Eur J Oral Sci. 1998;106:963–70. 10.1046/j.0909-8836.1998.eos106510.x. 9786327 10.1046/j.0909-8836.1998.eos106510.x

[CR4] Bloch-Zupan A, Huckert M, Stoetzel C, Meyer J, et al. Detection of a novel DSPP mutation by NGS in a population isolate in Madagascar. Front Physiol. 2016;2(7):70. 10.1186/1471-2350-11-23. 10.3389/fphys.2016.00070 PMC4773637 26973538

[CR5] de La Dure-Molla M, Phillipe Fournier B, Berdal A. Isolated dentinogenesis imperfecta and dentin dysplasia: revision of the classification. Eur J Hum Genet. 2015;23(4):445–51. 10.1038/ejhg.2014.159. 25118030 10.1038/ejhg.2014.159 PMC4666581

[CR6] Dong J, Gu T, Jeffords L, MacDougall M. Dentin phosphoprotein compound mutation in dentin sialophosphoprotein causes dentinogenesis imperfecta type III. Am J Med Genet. 2005;132(3):305–9. 10.1002/ajmg.a.30460. 10.1002/ajmg.a.30460 15690376

[CR7] Du Q, Cao L, Yan N, Kang S, et al. Identification of DSPP novel variants and phenotype analysis in dentinogenesis dysplasia shields type II patients. Clin Oral Investig. 2023;27(7):3885–94. 10.1007/s00784-023-05009-y. 37017752 10.1007/s00784-023-05009-y

[CR8] Du Q, Cao L, Pang C, Wu S, et al. Phenotype and molecular characterizations of a family with dentinogenesis imperfecta shields type II with a novel DSPP mutation. Ann Transl Med. 2021. 10.21037/atm-21-5369. 34988181 10.21037/atm-21-5369 PMC8667123

[CR9] Holappa H, Nieminen P, Tolva L, Lukinmaa PL, et al. Splicing site mutations in dentin sialophosphoprotein causing dentinogenesis imperfecta type II. Eur J Oral Sci. 2006;114(5):381–4. 10.1111/j.1600-0722.2006.00391.x. 17026502 10.1111/j.1600-0722.2006.00391.x

[CR10] Kantaputra PN, Chinadet W, Intachai W, Ngamphiw C, et al. Isolated dentinogenesis imperfecta with glass-like enamel caused by COL1A2 mutation. Am J Med Genet A. 2018;176(12):2919–23. 10.1002/ajmg.a.40501. 30152103 10.1002/ajmg.a.40501

[CR11] Kida M, Tsutsumi T, Shindoh M, Ikeda H, et al. De novo mutation in the DSPP gene associated with dentinogenesis imperfecta type II in a Japanese family. Eur J Oral Sci. 2009;117(6):691–4. 10.1111/j.1600-0722.2009.00683.x. 20121932 10.1111/j.1600-0722.2009.00683.x

[CR12] Kim JW, Nam SH, Jang KT, Lee SH, et al. A novel splice acceptor mutation in the DSPP gene causing dentinogenesis imperfecta type II. Hum Genet. 2004;115:248–54. 10.1007/s00439-004-1143-5. 15241678 10.1007/s00439-004-1143-5

[CR13] Kim JW, Hu JC, Lee JI, Moon SK, et al. Mutational hot spot in the DSPP gene causing dentinogenesis imperfecta type II. Hum Genet. 2005;116:186–91. 10.1007/s00439-004-1223-6. 15592686 10.1007/s00439-004-1223-6

[CR14] Kim YJ, Lee Y, Zhang H, Seymen F, et al. Translated mutant DSPP mRNA expression level impacts the severity of dentin defects. J Pers Med. 2022;12(6):1002. 10.3390/jpm12061002. 35743786 10.3390/jpm12061002 PMC9225647

[CR15] Lee SK, Hu JC, Lee KE, Simmer JP, et al. A dentin sialophosphoprotein mutation that partially disrupts a splice acceptor site causes type II dentin dysplasia. J Endod. 2008;34(12):1470–3. 10.1016/j.joen.2008.08.027. 19026876 10.1016/j.joen.2008.08.027 PMC2763612

[CR16] Lee SK, Lee KE, Jeon D, Lee G, et al. A novel mutation in the DSPP gene associated with dentinogenesis imperfecta type II. J Dent Res. 2009;88(1):51–5. 10.1177/0022034508328168. 19131317 10.1177/0022034508328168

[CR17] Lee KE, Kang HY, Lee SK, Yoo SH, et al. Novel dentin phosphoprotein frameshift mutations in dentinogenesis imperfecta type II. Clin Genet. 2011a;79(4):378–84. 10.1111/j.1399-0004.2010.01483.x. 20618350 10.1111/j.1399-0004.2010.01483.x

[CR18] Lee SK, Lee KE, Hwang YH, Kida M, et al. Identification of the DSPP mutation in a new kindred and phenotype-genotype correlation. Oral Dis. 2011b;17(3):314–9. 10.1111/j.1601-0825.2010.01760.x. 21029264 10.1111/j.1601-0825.2010.01760.x

[CR19] Lee SK, Lee KE, Song SJ, Hyun HK, et al. A DSPP mutation causing dentinogenesis imperfecta and characterization of the mutational effect. Biomed Res Int. 2013. 10.1155/2013/948181. 23509818 10.1155/2013/948181 PMC3591212

[CR20] Lee JW, Hong J, Seymen F, Kim YJ, et al. Novel frameshift mutations in DSPP cause dentin dysplasia type II. Oral Dis. 2019;25(8):2044–6. 10.1111/odi.13182. 31454439 10.1111/odi.13182 PMC7098805

[CR21] Lee Y, Kim YJ, Hyun HK, Lee JC, et al. Non-syndromic dentinogenesis imperfecta caused by mild mutations in COL1A2. J Pers Med. 2021;11(6):526. 10.3390/jpm11060526. 34201399 10.3390/jpm11060526 PMC8229930

[CR22] Li D, Du X, Zhang R, Shen B, et al. Mutation identification of the DSPP in a Chinese family with DGI-III and an up-to-date bioinformatic analysis. Genomics. 2012;99(4):220–6. 10.1016/j.ygeno.2012.01.006. 22310900 10.1016/j.ygeno.2012.01.006

[CR23] Li F, Liu Y, Liu H, Yang J. Phenotype and genotype analyses in seven families with dentinogenesis imperfecta or dentin dysplasia. Oral Dis. 2017;23(3):360–6. 10.1111/odi.12621. 27973701 10.1111/odi.12621

[CR24] Malmgren B, Lindskog S, Elgadi A, Norgren S. Clinical, histopathologic, and genetic investigation in two large families with dentinogenesis imperfecta type II. Hum Genet. 2004;114(5):491–8. 10.1007/s00439-004-1084-z. 14758537 10.1007/s00439-004-1084-z

[CR25] McKnight DA, Hart PS, Hart TC, Hartsfield JK, et al. A comprehensive analysis of normal variation and disease-causing mutations in the human DSPP gene. Hum Mutat. 2008a;29(12):1392–404. 10.1002/humu.20783. 18521831 10.1002/humu.20783 PMC5534847

[CR26] McKnight DA, Simmer JP, Hart PS, Hart TC, et al. Overlapping DSPP mutations cause dentin dysplasia and dentinogenesis imperfecta. J Dent Res. 2008b;87(12):1108–11. 10.1177/154405910808701217. 19029076 10.1177/154405910808701217 PMC2596760

[CR27] Nieminen P, Papagiannoulis-Lascarides L, Waltimo-Siren J, Ollila P, et al. Frameshift mutations in dentine phosphoprotein and dependence of dentine disease phenotype on mutation location. J Bone Miner Res. 2011;26(4):873–80. 10.1002/jbmr.276. 20949630 10.1002/jbmr.276

[CR28] Ouzzani H, Fedorowicz EA, et al. Rayyan – a web and mobile app for systematic reviews. Syst Rev. 2016. 10.1186/s13643-016-0384-4. 27919275 10.1186/s13643-016-0384-4 PMC5139140

[CR29] Page MJ, McKenzie JE, Bossuyt PM, Boutron I, et al. The PRISMA 2020 statement: an updated guideline for reporting systematic reviews. Syst Rev. 2021;10(1):89. 10.1136/bmj.n71. 33781348 10.1186/s13643-021-01626-4 PMC8008539

[CR30] Porntaveetus T, Osathanon T, Nowwarote N, Pavasant P, et al. Dental properties, ultrastructure, and pulp cells associated with a novel DSPP mutation. Oral Dis. 2018;24(4):619–27. 10.1111/odi.12801. 29117466 10.1111/odi.12801

[CR31] Porntaveetus T, Nowwarote N, Osathanon T, Theerapanon T, et al. Compromised alveolar bone calls in a patient with dentinogenesis imperfecta caused by DSPP mutation. Clin Oral Investig. 2019;23(1):303–13. 10.1007/s00784-018-2437-7. 29679229 10.1007/s00784-018-2437-7

[CR32] Rajpar MH, Koch MJ, Davies RM, Mellody KT, et al. Mutation of the signal peptide region of the bicistronic gene DSPP affects translocation to the endoplasmic reticulum and results in defective dentine biomineralization. Hum Molec Genet. 2002;11(21):2559–65. 10.1093/hmg/11.21.2559. 12354781 10.1093/hmg/11.21.2559

[CR33] Shields ED, Bixler D, el Kafrawy A. proposed classification for heritable human dentine defects with a description of a new entity. Arch Oral Biol. 1973;18(4):543–53. 4516067 10.1016/0003-9969(73)90075-7

[CR34] Simmer JP, Zhang H, Moon SJ, Donnelly LA, et al. The modified Shields classification and 12 families with defined DSPP mutations. Genes. 2022;13(5):858. 10.3390/genes13050858. 35627243 10.3390/genes13050858 PMC9141616

[CR35] Song Y, Wang C, Peng B, Ye X, et al. Phenotypes and genotypes in 2 DGI families with different DSPP mutations. Oral Surg Oral Med Oral Pathol Oral Radiol Endod. 2006;102(3):360–74. 10.1016/j.tripleo.2005.06.020. 16920545 10.1016/j.tripleo.2005.06.020

[CR36] Song YL, Wang CN, Fan MW, Su B, et al. Dentin phosphoprotein frameshift mutations in hereditary dentin disorders and their variation patterns in normal human population. J Med Genet. 2008;45(7):457–64. 10.1136/jmg.2007.056911. 18456718 10.1136/jmg.2007.056911

[CR37] Suzuki S, Sreenath T, Haruyama N, Honeycutt C, et al. Dentin sialoprotein and dentin phosphoprotein have distinct roles in dentin mineralization. Matrix Biol. 2009;28(4):221–9. 10.1016/j.matbio.2009.03.006. 19348940 10.1016/j.matbio.2009.03.006 PMC2758621

[CR38] Taleb K, Laurisden E, Daugaard-Jensen J, Nieminen P, et al. Dentinogenesis imperfecta type II- genotype and phenotype analyses in three Danish families. Mol Genet Genomic Med. 2018;6(3):339–49. 10.1002/mgg3.375. 29512331 10.1002/mgg3.375 PMC6014476

[CR39] Udomchaiprasertkul W, Kuptanon C, Porntaveetus T, Shotelersuk V. A family with homozygous and heterozygous pGly337Ser mutations in COL1A2. Eur J Med Genet. 2020;63(6):103896. 10.1016/j.ejmg.2020.103896. 32081708 10.1016/j.ejmg.2020.103896

[CR40] Wang HY, Hou YN, Cui YX, Huang YF, et al. A novel splice site mutation in the dentin sialophosphoprotein gene in a Chinese family with dentinogenesis imperfecta type II. Mutat Res. 2009;662(1):22–7. 10.1016/j.mrfmmm.2008.11.019. 19103209 10.1016/j.mrfmmm.2008.11.019

[CR41] Witkop CJ. Hereditary defects in enamel and dentin. Acta Genet Stat Med. 1957;7(1):236–9. 10.1159/000150974. 13469154 10.1159/000150974

[CR42] Xiao S, Yu C, Chou X, Yuan W, et al. Dentinogenesis imperfecta 1 with or without progressive hearing loss is associated with distinct mutations in DSPP. Nat Genet. 2001;27(2):201–4. 10.1038/84848. 11175790 10.1038/84848

[CR43] Yamakoshi Y. Dentin sialophosphoprotein (DSPP) and dentin. J Oral BioSci. 2008;50(1):33–44. 10.2330/joralbiosci.50.33. 20037676 10.2330/joralbiosci.50.33 PMC2797732

[CR44] Yang J, Kawasaki K, Lee M, Reid B, et al. The dentin phosphoprotein repeat region and inherited defects of dentin. Mol Genet Genomic Med. 2016;4(1):28–38. 10.1002/mgg3.176. 26788535 10.1002/mgg3.176 PMC4707025

[CR45] Yuan M, Zheng X, Xue Y, He Z, et al. A novel DSPP frameshift mutation causing dentin dysplasia type 2 and disease management strategies. Oral Dis. 2023;29(2):855. 10.1111/odi.14494. 36597617 10.1111/odi.14494

[CR46] Zeng Y, Pan Y, Mo J, Ling Z, et al. Case report: a novel COL1A1 missense mutation associated with dentinogenesis imperfecta type I. Front Genet. 2021;12: 699278. 10.3389/fgene.2021.699278. 34249109 10.3389/fgene.2021.699278 PMC8260930

[CR47] Zhang X, Zhao J, Li C, Gao S, et al. DSPP mutation in dentinogenesis imperfecta Shields type II. Nat Genet. 2001;27(2):151–2. 10.1038/84765. 11175779 10.1038/84765

[CR48] Zhang X, Chen L, Liu J, Zhao Z, et al. A novel DSPP mutation is associated with type II dentinogenesis imperfects in a Chinese family. BMC Med Genet. 2007;8:1–6. 10.1186/1471-2350-8-52. 17686168 10.1186/1471-2350-8-52 PMC1995191

[CR49] Zhang J, Wang J, Ma Y, Du W, et al. A novel splicing mutation alters DSPP transcription and leads to dentinogenesis imperfecta type II. PLoS ONE. 2011;6(11): e27982. 10.1371/journal.pone.0027982. 22125647 10.1371/journal.pone.0027982 PMC3220712

[CR50] Zhang Z, Huang G, Huang Y, Liu S, et al. Novel dentin sialophosphoprotein gene frameshift mutations affect dentin mineralization. Arch Oral Biol. 2023;1: 105701. 10.1016/j.archoralbio.2023.105701. 10.1016/j.archoralbio.2023.105701 37084484

